# GREM1 deficiency induced bone marrow adipose niche promotes B‐cell acute lymphoblastic leukemia disease progression

**DOI:** 10.1002/ijc.35418

**Published:** 2025-04-26

**Authors:** Lili Song, Rui Zhang, Liya Pan, Qiang Mi, Yi Yang, Xiang Wang, Yani Ma, Shuhong Shen, Benshang Li, Yanxin Li, Li Hong

**Affiliations:** ^1^ Department of Clinical Nutrition, Shanghai Children's Medical Center Shanghai Jiao Tong University School of Medicine Shanghai China; ^2^ Department of Hematology & Oncology, Key Laboratory of Pediatric Hematology and Oncology Ministry of Health Shanghai Children's Medical Center, Shanghai Jiao Tong University School of Medicine Shanghai China; ^3^ Pediatric Translational Medicine Institute, Shanghai Children's Medical Center Shanghai Jiao Tong University School of Medicine Shanghai China

**Keywords:** adipogenic differentiation, bone marrow microenvironment, childhood B‐cell acute lymphocytic leukemia, dyslipidemia, GREM1

## Abstract

Relapse and disease progression are the primary causes of treatment failure and subsequent mortality in children with B‐cell acute lymphocytic leukemia (B‐ALL). At diagnosis and during treatment, dyslipidemia and the bone marrow adipose microenvironment are commonly observed in pediatric leukemia. However, the intricate connection between these factors and disease progression remains largely unexplored. We found that abnormal triglyceride accumulation increased the risk of death. Further investigation into the adipogenic potential of BM‐MSCs revealed a correlation between higher adipogenicity and elevated serum TG levels, which subsequently led to the rapid proliferation of leukemia cells and heightened the risk of post‐relapse mortality. Through RNA sequencing, Gremlin1 (GREM1) was identified as an important factor affecting adipogenicity. Silencing of *GREM1* in BM‐MSCs induced adipogenic differentiation, partly through the BMP/SMAD signaling pathway. In an in vitro co‐culture model, shGREM1‐MSCs promoted B‐ALL cell proliferation and induced drug resistance to dexamethasone, while increasing sensitivity to L‐asparaginase. Furthermore, GREM1‐deficient BM‐MSCs promoted B‐ALL disease progression in xenograft models. This study provides new insights into overcoming drug resistance, relapse, and death by elucidating the novel mechanism by which GREM1 deficiency induces adipogenic differentiation of BM‐MSCs and promotes B‐ALL disease progression.

## Graphical abstract

What's new?

Too often, children treated for B‐cell acute lymphocytic leukemia (B‐ALL) experience relapse. Here, the authors investigate the role of dyslipidemia in leukemia progression. Higher adipogenicity in bone marrow mesenchymal stem cells, and increased triglyceride accumulation, are associated with higher risk of death. This study showed that silencing of GREM‐1 induced adipogenicity, promoted B‐ALL disease progression and contributed to dexamethasone resistance. These results show that GREM‐1 deficiency can be considered a clinical indicator of poor prognosis, and potentially suggests a new therapeutic strategy.

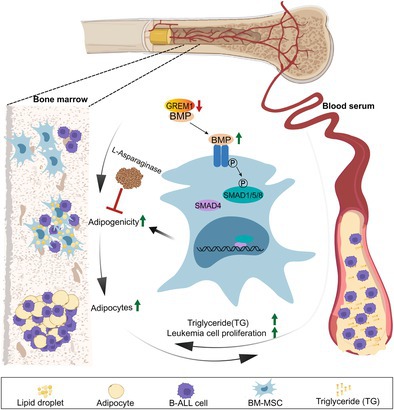

**Abbreviations**
ALL: Acute lymphoblastic leukemia
AML: Acute myelocytic leukemia
apoA1: Apolipoprotein A1
apoB: Apolipoprotein B
B‐ALL: B cell acute lymphoblastic leukemia
BM: Bone marrow
BM‐Ads: Bone marrow adipocytes
BM‐MSCs: Bone marrow mesenchymal stem cells
BM niche: Bone marrow microenvironment
BMP: Bone morphogenetic protein
CCCG‐ALL‐2015: Chinese Children's Cancer Group ALL‐2015
CEBPA: CCAAT/enhancer binding protein‐alpha
CR: Complete response
CREB3L3: cAMP‐Responsive Element‐Binding Protein 3‐like Protein 3
CXCR4: C‐X‐C Motif Chemokine Receptor 4
DXMS: Dexamethasone
EMT: Epithelial‐mesenchymal transition
FABP4: Fatty acid Binding Protein 4
FFA: Free fatty acid
GREM1: Gremlin‐1
HDL‐C: High density lipoprotein cholesterol
High AD: High‐efficiency adipogenic differentiation
L‐ASP: L‐asparaginase
LDL‐C: Low density lipoprotein cholesterol
Low AD: Low‐efficiency adipogenic differentiation
MRD: Minimal residual disease
ND: Normal donors
PCA: Principal‐component analysis
PPARG: Peroxisome proliferator‐activated receptor γ
RNA‐seq: RNA sequencing
RT‐PCR: Real‐time quantitative PCR
SFRP: Secreted frizzled‐related proteins
T‐ALL: T cell acute lymphoblastic leukemia
TG: Triglyceride
TGFβ: Transforming growth factor‐β
WB: Western blot
WNT16: Wingless‐type MMTV integration site family, member 16

## 1 INTRODUCTION

B‐cell acute lymphoblastic leukemia (B‐ALL) is the most common type of cancer in children.^1^, ^2^ Although treatment outcomes for childhood B‐ALL have improved significantly, approximately 20% of children develop refractory or relapsed disease after achieving complete remission (CR), leading to high mortality.^3^, ^4^ Tumor metabolic reprogramming plays a crucial role in chemoresistance and tumor progression. Abnormal lipid metabolism is present in children with ALL at diagnosis and prior to therapy,^5^, ^6^, ^7^ which could potentially result in bone marrow failure.^8^ Apart from the observed increase in intracellular lipid synthesis in tumor cells to accommodate the increased demand for fatty acids for membrane biogenesis and energy production, there have also been reports of upregulation of extracellular lipid uptake. Both the bone marrow and peripheral blood are major sources of extracellular lipid uptake. However, studies on the relationship between the adipocyte‐marrow niche, dyslipidemia, and treatment outcome in children with B‐ALL have yielded inconsistent results.

The adipocyte bone marrow niche serves as a protective environment for leukemia cells and may provide them with important survival signals.^9^ Within this niche, bone marrow adipocytes (BM‐Ads) derived from mesenchymal stem cells (MSC) are the most abundant stromal component, accounting for 50%–70% of the total marrow volume and approximately 5%–10% of body fat.^10^ Hormonal and metabolic changes that occur during the progression of leukemia have been found to affect the bone marrow microenvironment. These changes disrupt the balance of osteogenic and adipogenic differentiation of BM‐MSCs. Recent studies have found that human Acute myeloid leukemia (AML) can specifically disrupt the adipocytic niche in BM,^11^ which is inversely correlated with hematopoiesis that may impact leukemia cell survival.^12^ Additionally, despite their supportive function, adipocytes also have a direct role in negatively regulating hematopoiesis.^13^, ^14^ BM‐Ads have been found to negatively affect T‐ALL proliferation and mediate chemoresistance in vitro and in vivo.^15^ However, the role of adipocytes in the BM niche is still multifaceted and largely unknown in B‐ALL.

As an endogenous antagonist of bone morphogenetic protein (BMP), Gremlin1 (GREM1) is overexpressed in various human tumors, including sarcoma and carcinomas of the lung, ovary, kidney, breast, colon, and pancreas.^16^, ^17^ GREM1 has also been shown to inhibit BMP binding at the plasma membrane.^18^, ^19^ The BMP family is an essential signaling protein that promotes MSCs osteogenic differentiation. A recent study suggested that MSCs delivering gremlin1 promoted the tumor epithelial‐mesenchymal transition (EMT) in esophageal squamous cell carcinoma^20^ and aristolochic acid nephropathy.^21^ Furthermore, *GREM1* has been found to be one of the most upregulated genes in the tumor microenvironment.^22^ These findings demonstrate the important role of GREM1 in cancer development, especially in the microenvironmental niches. However, the role and mechanism of GREM1 in BM‐MSCs of B‐ALL remain poorly understood.

This study aimed to investigate the correlation between extracellular lipid changes and B‐ALL disease progression. Furthermore, we identified GREM1 as an important factor in the regulation of adipogenic differentiation of BM‐MSCs.

## 2 MATERIALS AND METHODS

### 2.1 Patients

The retrospective study analyzed serum lipid levels in a cohort of children aged 4 months to 17 years who were newly diagnosed with pediatric B‐ALL at the Shanghai Children's Medical Center (SCMC) in China, between April 18, 2018 and November 26, 2020. Patients were treated on the Chinese Children's Cancer Group ALL‐2015 (CCCG‐ALL‐2015) protocol or CCCG‐ALL‐2020 protocol.^23^ Patients without serum lipid data at diagnosis were excluded from the study. The high TG level was defined as TG ≥2.26 mmol/L, following the guidelines of the American Heart Association. Treatment response was assessed by measuring MRD levels during remission induction at Days 19 and 46. Serum lipid data from patients aged 2 to 13 years diagnosed with obesity between May 12, 2017 and December 12, 2020, and the normal group aged 5 months to 18 years from children's physical examination between May 29, 2017 and November 23, 2020, were collected. Data analysis was conducted from July 1 to November 20, 2022 in a blind manner.

### 2.2 MSC preparation and adipogenic differentiation

Human bone marrow mesenchymal stem cells (BM‐MSCs) were isolated from the marrow of 17 B‐ALL patients at diagnosis, 21 at relapse, and 6 normal donors at SCMC between November 12, 2020 and July 2021. The cells were cultured in Minimum Essential Medium (MEM) α (Thermo Fisher, Waltham, MA) with 10% fetal bovine serum (Thermo Fisher, Waltham, MA) at 37°C and 5% CO2. The medium was replaced every 3 days. BM‐MSCs at passages 3–10 were used for all experiments. For adipogenic differentiation, BM‐MSCs at passage 3 were seeded at a density of 1 × 10^5^ per well in a 48‐well plate in adipogenesis induction medium (Cyagen Biosciences Inc., Shanghai, China) following the manufacturer's instructions. Lipid staining was performed using Oil Red O staining by incubating with Oil Red O solution for 30 min, as stated in the manual. Images were captured using Leica microscopy (Leica, Wetzlar, Germany). The adipogenic efficiency is represented by the percentage area of Oil Red staining measured with Image J Software (NIH). The cut‐off value was set at 10%, calculated as the geometric mean of the adipogenic efficiency of all samples. High‐AD was defined as adipogenic efficiency ≥10%, while low‐AD was defined as adipogenic efficiency <10%.

### 2.3 Flow cytometry analysis

Various BM‐MSCs (2 × 10^5^) were collected at passage 3 and stained with the CD105‐APC, CD73‐V450, CD90‐FITC, CD34‐PerCP, CD24‐APC, CD36‐FITC, CD38‐V450, and CD45‐V500 (BD, Franklin Lakes, NJ). The Flow Cytometry data were analyzed with primary gating to exclude debris using the FSC‐A/SSC‐A gate, the FSC‐A/FSC‐H gate to keep only single cells, and isotype IgG were used as a negative control for flow cytometry gating.

### 2.4 qRT‐PCR analysis and RNA sequencing

Total RNA was extracted using the RNeasy plus kit (Qiagen, MD) in accordance with the manufacturer's protocol. Real‐time PCR was performed using the SYBR Green PCR Master Mix on a 7500 Fast Real‐Time PCR System (Thermo Fisher, Waltham, MA). The primers used are listed in Table S4. RNA sequencing was performed by the High‐Throughput Genomics Group at Mingma technologies (Shanghai, China). After the library was constructed, Qubit was used to detect the concentration of the library, and Agilent fragment analyzer was used to detect the fragment length to ensure the quality of the library. After the library was qualified, the Illumina Novaseq 6000 sequencing platform was used for PE150 sequencing. PE150(Pair end 150 bp) refers to a high‐throughput two‐end sequencing strategy, with each end measuring 150 bp. The sequencing coverage and quality statistics for each sample are summarized in Table S5.

### 2.5 Stable gene knockdown and overexpression

Lentiviral shRNAs were utilized to downregulate the expression of gremlin1 in BM‐MSCs. The shRNA sequences, namely shRNA#1, shRNA#2, and shRNA#3, were 5'‐ATTTGCGCTCCGTCACATGC‐3', 5'‐TCGTTGCATATCCATCGATT‐3', and 5'‐GCTTAAGCAGACCATCCACG‐3', respectively. These sequences were then cloned into pLKO.1‐puro. For the cloning of human *GREM1* coding regions, pLenti6‐blast Vector (Addgene, Watertown, MA) was used, and the cloning was validated through DNA sequencing. The constructs were transfected into HEK‐293 T cells with packaging plasmids psPAX2 and pMD2G using the calcium phosphate method to generate a replication‐defective virus. After 48 h, the supernatant was harvested and concentrated using a 100 kDa column (Millipore Sigma, Burlington, MA). BM‐MSCs were then transduced with the virus in the presence of 8 μg/mL polybrene (Sigma Aldrich, Burlington, MA). The medium was changed 24 h post‐infection, and puromycin or blasticidin‐resistant cells were selected by adding 1 μg/mL puromycin or 10 μg/mL blasticidin for a duration of 5 days. The stable gene knockdown and overexpression experiments were conducted using three BM‐MSCs that were isolated from the marrow of three normal donors (N01, N02, N03), which were also used in the other experiments of this study. As a control, cells were transfected with a scramble control.

### 2.6 Cell line culture

The human B‐ALL cell lines NALM‐6 (RRID:CVCL_0092) and RS4;11 (RRID:CVCL_0093), as well as the HEK‐293 T (RRID:CVCL_0063) cells, were obtained from ATCC (Gaithersburg, MA. USA). NALM‐6 and RS4;11 cells were cultured in 10% FBS/RPMI‐1640 medium and 1% penicillin–streptomycin. HEK‐293 T cells were cultured in 10% FBS/DMEM medium. All cells were incubated at 37°C in a 5% CO_2_ environment. All cell lines were authenticated using short tandem repeat (STR) profiling within the last 3 years. All experiments were performed with mycoplasma‐free cells.

### 2.7 Co‐culture of BM‐MSCs and the B‐ALL cells

0.2 × 10^6^ BM‐MSCs and 0.2 × 10^6^ B‐ALL cells were co‐cultured in a 12 well plate. To determine the number of B‐ALL cells at various time points, we first counted the total cell population. The cells were then stained with anti‐CD19 antibody, and the percentage of CD19‐positive cells was assessed using flow cytometry. Based on these percentages, we specifically calculated the number of B‐ALL cells (NALM‐6 cells).

### 2.8 Fatty acid uptake and immunofluorescence staining

BM‐MSCs and NALM‐6 cells were treated with Oleic acid (Sigma Aldrich, Burlington, MA) and stained with BODIPY 493/503 (Glpbio, Montclair, CA) for microscopy. Quantification was performed using flow cytometry, following the manufacturer's protocol.^24^ To assess drug effects on fatty acid uptake, NALM‐6 cells were exposed to dexamethasone (1 μM) and L‐asparaginase (1 IU/mL), either in combination or alone, and preincubated for 1 h. The uptake assay for Lauric acid (Dodecanoic acid) was conducted using the Screen Quest™ Fluorimetric Fatty Acid Uptake Assay Kit (AAT Bioquest, Sunnyvale, CA), following the manufacturer's instructions. The data were plotted in flow cytometry overlay histograms with Kaluza analysis software (Kaluza Analysis 2.1, Beckman Coulter). The method is equally applicable to BM‐MSCs.

### 2.9 Lipid metabolomics

NALM‐6 cells were treated with Oleic acid (Sigma Aldrich, Burlington, MA) or lauric acid (AAT Bioquest, Sunnyvale, CA) in the presence of culture supernatant of BM‐MSCs for 24 h. Patient PBMCs were isolated using Ficol‐Hypaque (Sigma) gradient centrifugation. The collected cells were then analyzed to identify metabolic alterations among the different groups. Metabolomics and lipidomics analyses were performed using liquid chromatography‐mass spectrometry (LC–MS) at the Shanghai Institute of Nutrition and Health (SINH), Chinese Academy of Sciences (CAS), Shanghai, China.

### 2.10 Western blotting

Western blotting was performed as previously described.^25^ The primary antibodies used were against gremlin‐1 and β‐actin (Santa Cruz, CA), P‐SMAD1/5/9 and SMAD1 (CST, Danvers, MA). Immunoblots were analyzed using the Odyssey system (LI‐COR, Lincoln, NE). Grayscale values of proteins were evaluated by ImageJ (https://imagej.nih.gov/ij/).

### 2.11 Cell viability

Cell viability was determined by using the Cell Titer‐Glo Luminescent kit (Promega, Madison, WI) according to the manufacturer's instructions as previously described. NALM‐6 cells were seeded at a density of 10,000 cells per well in 96‐well plates and treated with different serial dilutions of Dexamethasone or L‐asparaginase for 72 h. Subsequently, 50 μL of Cell Titer‐Glo Reagents were added to each well and mixed for 10 min, before measuring the luminescent signal using a microplate reader (Biotek, Winooski, VT). All experiments were performed in triplicate, and the results were calculated as the mean ± SD. Comparisons between multiple groups were performed using ANOVA with the Bonferroni test.

### 2.12 Tumorigenesis studies

Female mice with Severe combined immunodeficiency (SCID), aged 4–6 weeks, were used for this study. The mice were obtained from SLAC, Shanghai, China. They were randomly divided into groups of five mice each. A total of 1 × 10^6^ NALM‐6 cells mixed with 2 × 10^5^ BM‐MSCs were injected into the recipients through the tail vein. BM‐MSCs characterized by high GREM1 expression and low adipogenic efficiency are designated as the ‘Low AD’ group (N04, P02, P06), while those with low GREM1 expression and high adipogenic efficiency are categorized as the ‘High AD’ group (P03, R02, R03). Mice were euthanized when ALL symptoms developed.

## 3 STATISTICS

The lipid formation efficiency was analyzed using Image J software (Image J, NIH). Statistical analysis was performed with GraphPad Prism 9.0 software. The measured data results are presented as mean ± SD. Statistical differences among independent samples were analyzed using Student's *t* test, Mann–Whitney test, Chi‐square test, and Fisher exact test. Comparisons between multiple groups were performed using ANOVA with the Bonferroni test. The significant statistical differences are indicated as follows: ns (not significant) for *p* > 0.05, * for 0.01 < *p* < 0.05, ** for 0.001 < *p* < 0.01, *** for 0.0001 < *p* < 0.001, and **** for *p* < 0.0001.

## 4 RESULTS

### 4.1 Impact of dyslipidemia on the treatment of childhood B‐cell acute lymphoblastic leukemia

Metabolic dysfunction plays a major role in survivors of childhood ALL, and lipid abnormalities lead to an increased risk of morbidity and mortality throughout their life.^26^, ^27^

**FIGURE 1:**
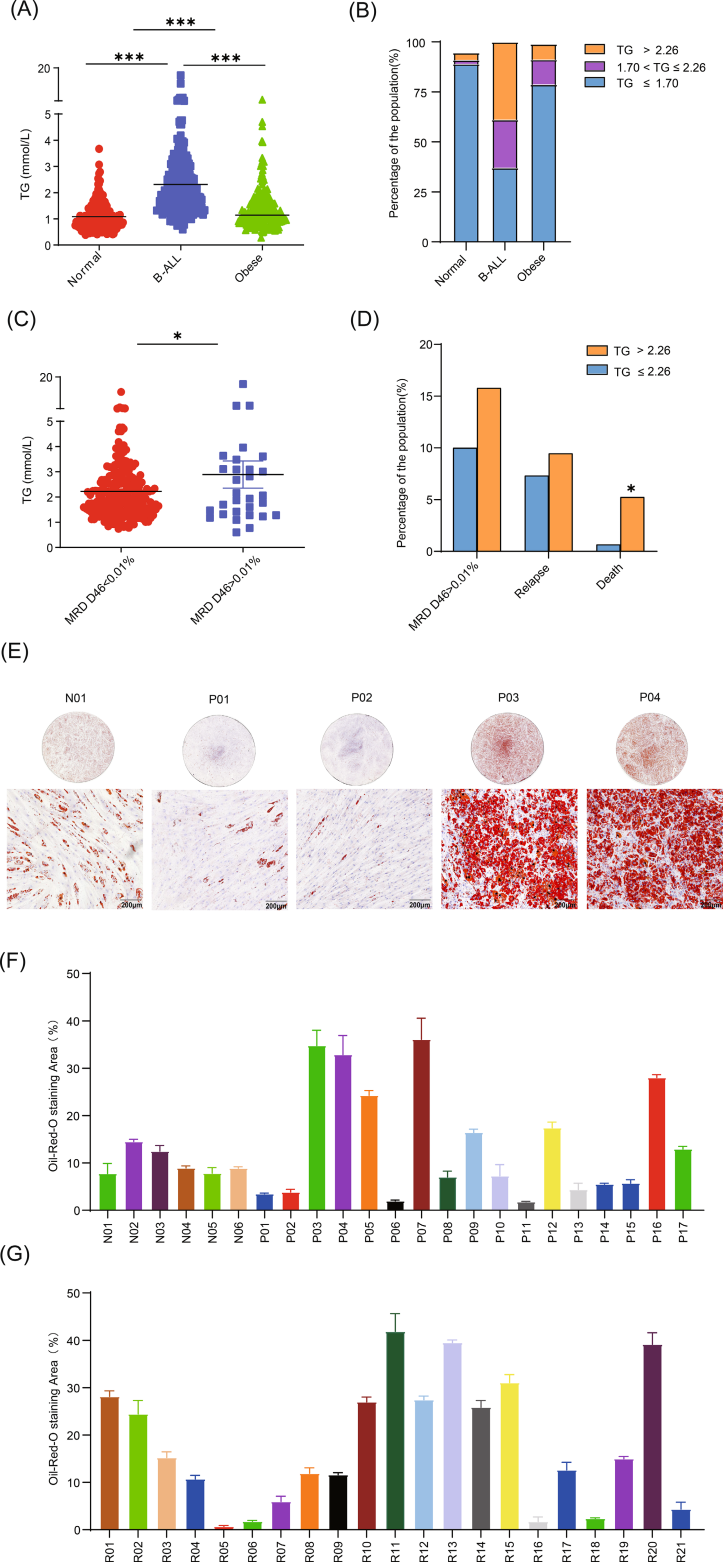
Dyslipidemia is closely associated with increased risk of death in childhood B‐ALL, and the efficiency of BM‐MSCs adipogenic differentiation in children with B‐ALL is heterogeneous. (A) Scatter plot of serum triglycerides (mmol/L) in normal group, B‐ALL, and obese patients. The figures were made using GraphPad Prism 9.0. Comparisons were calculated using two‐sided Mann–Whitney tests, ***0.0001 < *p* < 0.001. (B) Percentage distribution of TG level ≤1.7 mmol/L,1.70 < TG≤2.26 and TG level >2.26 mmol/L) among normal group, B‐ALL, and obese patients. (C) Scatter plot of serum triglycerides (mmol/L) in both group (MRD D46 < 0.01% and MRD D46 >0.01%), Comparisons were calculated using two‐sided Mann–Whitney tests, *0.01 < *p* < 0.05. (D) Percentage distribution of MRD D46, Relapse and Death of B‐ALL patients in both groups (TG level ≤2.26 mmol/L and TG level >2.26 mmol/L). (p‐values were generated from Chi‐square test/Fisher exact test, *0.01 < *p* < 0.05). (E) Oil Red O staining of lipid droplets in BM‐MSCs during adipogenic differentiation. Top: A full field of view image, and bottom: 40×, scale bars 200 μm. (F) Relative efficiency of adipogenic differentiation of patients at diagnosis and normal donors by quantification of Oil Red O staining. (G) Relative efficiency of adipogenic differentiation of patients at relapse by quantification of Oil Red O staining.

We performed a retrospective statistical analysis of lipid panel that defines dyslipidemia at diagnosis in 245 children diagnosed with B‐ALL (60.8% males) with a median age of 5.3 years Triglyceride levels (TG) were categorized as follows: levels below 1.7 mmol/L were considered within the normal range, those between 1.7 and 2.25 mmol/L were defined as borderline elevated, and levels ≥2.26 mmol/L were classified as elevated triglycerides. The level of triglycerides in B‐ALL is significantly higher than the normal group and obese patients (Figure 1A), whereas the level of total cholesterol, apolipoprotein A1(apoA1), high‐ and low‐density lipoprotein cholesterol (HDL and LDL) is lower, and Apolipoprotein B (apoB) and Lipoprotein (a) showed no difference (Figure S1A–C). Among children undergoing routine health examinations, 171 individuals (89.06%) exhibited triglyceride levels below 1.7 mmol/L, whereas only nine children (3.67%) had levels exceeding 2.26 mmol/L. In the obese children group, 78.78% had triglyceride levels below 1.7 mmol/L, and 7.76% had levels above 2.26 mmol/L. In contrast, in the B‐ALL patient group, only 91 cases (37.14%) had triglyceride levels below 1.7 mmol/L, while 95 cases (38.78%) had levels above 2.26 mmol/L (Figure 1B). Furthermore, age and sex have no significant effect on TG levels (Figure S1D–F and Table S1). Patients with a minimal residual disease (MRD) burden >0.01% at end induction have an increased risk of relapse.^28^ To investigate the impact of increased TG levels at diagnosis on chemotherapy, we evaluated the treatment response via MRD during remission induction therapy on Day 46. Patients with D46 MRD >0.01% showed higher triglyceride levels (Figure 1C). Among the 95 patients with high TG levels (>2.26 mmol/L), 9.47% (9/95) of patients experienced relapse and 5.26% (5/95) death at the end of the study. In contrast, among the patients with low TG levels (≤2.26 mmol/L), 7.33% (11/150) experienced relapse and 0.67% (1/150) death (Figure 1D and Table S1). Apart from TG abnormalities, bacterial/viral infections that cannot be effectively controlled led to septic shock and, in some cases, organ failure, ultimately resulting in death for four patients. Two patients with central nervous system (CNS) died after treatment failure. Through multivariate logistic regression analysis, we identified TG levels and infection as significant factors affecting mortality (Table S2). These data support that the abnormal TG accumulation in serum is associated with drug resistance and significantly increased risk of death in childhood B‐ALL.

### 4.2 Heterogeneity in adipogenic differentiation of bone marrow mesenchymal stem cells

**FIGURE 2:**
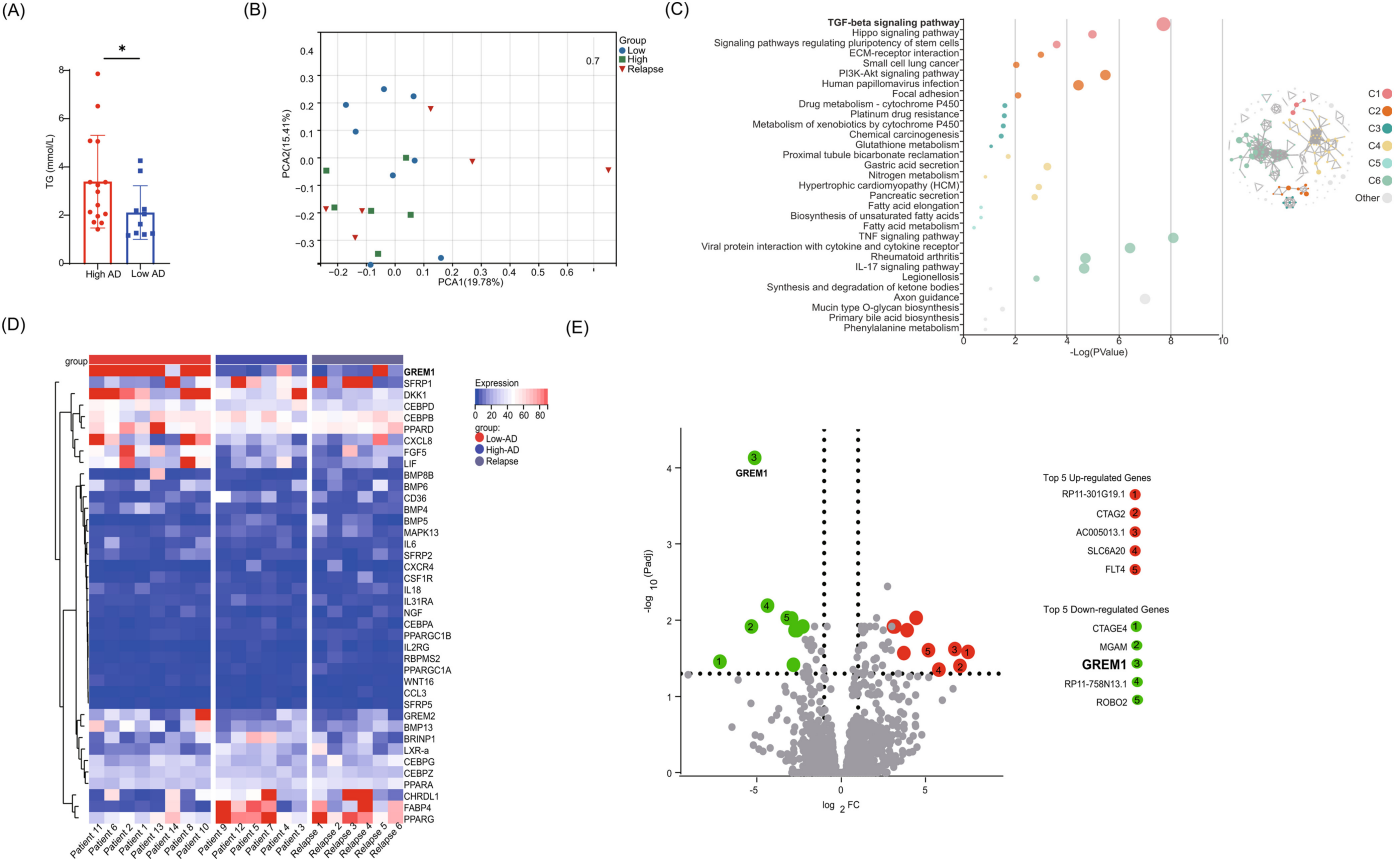
GREM1 is a key regulator of BM‐MSCs adipogenic differentiation. (A) Correlation between the efficiency of BM‐MSCs adipogenic differentiation and triglyceride levels in serum. The figure was made using GraphPad Prism 9.0. Comparisons were calculated using two‐sided Mann–Whitney tests, ns *p* > 0.05, *0.01 < *p* < 0.05. (B) A principal component analysis (PCA) was performed on the RNAseq dataset of 20 BM‐MSC samples on the Sangerbox. (C) GO and KEGG pathway enrichment analysis. Left: Bubble plot of GO/KEGG enrichment analysis, and right: Network visualization of GO/KEGG enrichment analysis. (D) Clustering heat map of adipogenic differentiation‐related differential genes in high‐AD, low‐AD, and relapse populations. (E) A volcano map of the differential genes in the RNAseq dataset.

Altered lipid metabolism can lead to bone marrow failure. Adipocytes derived from BM‐MSCs are the main source of fatty acids required for leukemia cell growth and contribute to the formation of protective BM niches. In this study, BM‐MSCs obtained from 6 normal donors (ND), 17 children with B‐ALL at diagnosis, and 21 relapsed patients were assayed for their ability to differentiate into adipocytes. After three rounds of induction in adipogenic medium at passage 3, B‐ALL MSCs exhibited varying amounts of Oil Red O stained cells compared to ND‐MSCs (Figure 1E–G and Table 1). Among the BM‐MSCs at diagnosis, 47.06% (8/17) showed a high efficiency of adipogenic differentiation (High AD, ≥10%), while 71.34% (15/21) of the BM‐MSCs at relapse exhibited high AD. We further analyzed the changes in BM‐MSCs before and after adipogenic differentiation. The BM‐MSCs were immunophenotyped via flow cytometry analysis of surface antigen expression, specifically MSC defining markers (CD45‐CD34‐CD73 + CD90 + CD105+); there were no significant differences in the expression of MSC defining markers between ND‐MSCs and B‐ALL‐MSCs (Figure S2A,B). However, during adipogenic differentiation, we observed a notable upregulation of CD36 and downregulation of CD73 (Figure S2C,D). CD36, known for its role as a scavenger receptor for various ligands, has been demonstrated to influence proliferation and the expression of lipogenic genes during adipogenesis.^29^ The metabolism of NAD+ through ectoenzymatic pathways relies on adenosine‐generating enzymes CD38 and CD73, potentially contributing to tumor development, including leukemia.^30^, ^31^ This suggests a potential role of CD36 and CD73 in promoting leukemia development within such an adipocytic microenvironment. The lipid panel screening data was analyzed in conjunction with the efficiency of adipogenic differentiation. Interestingly, we found that BM‐MSCs with high efficiency of adipogenic differentiation (High‐AD) were usually correlated with elevated levels of triglycerides in the serum (*p* = 0.0484, see Figure 2A and Table 1).

**TABLE 1:** The information of patients at diagnosis and relapse.

Group	Patient ID	TG (mmol/L)	Adipogenic efficiency (%)	Clinical outcome
High‐AD	P07	7.86	36.10	CR
High‐AD	P03	2.97	34.78	CR
High‐AD	P04	1.76	32.89	CR
High‐AD	P05	3.37	24.27	CR
High‐AD	P12	6.52	17.42	CR
High‐AD	P09	3.37	16.44	CR
Low‐AD	P10	2.05	7.28	CR
Low‐AD	P08	1.16	7.03	CR
Low‐AD	P14	4.26	5.50	Death
Low‐AD	P13	2.19	4.35	CR
Low‐AD	P02	1.2	3.82	CR
Low‐AD	P01	2.25	3.48	CR
Low‐AD	P06	1.24	1.96	CR
Low‐AD	P11	1.26	1.78	CR
High‐AD	R01	5.06	28.15	Relapsed after 9 years, Death
High‐AD	R02	1.68	24.43	Relapsed after 3 years, Death
High‐AD	R03	1.71	15.23	Relapsed after 2 years, Death
High‐AD	R04	2.42	10.7	Relapsed after 5 years, CR
Low‐AD	R05	2.05	0.69	Relapsed after 3 years, CR
Low‐AD	R06	2.05	1.74	Relapsed after 4 years, CR

These data suggest that the abnormal adipogenic differentiation of BM‐MSCs may be associated with the abnormal accumulation of lipids at diagnosis, especially triglycerides. the abnormal TG accumulation in serum is associated with drug resistance and significantly increased risk of death in childhood B‐ALL.

### 4.3 Key regulators of BM‐MSCs adipogenic differentiation

To investigate potential regulators of adipogenic differentiation of BM‐MSCs, we performed RNA sequencing (RNA‐seq) to further characterize the molecular identity of high‐AD and low‐AD populations (Table 1). The principal‐component analysis (PCA) results showed consistency between the two groups (Figure 2B), while the functional clustering analysis revealed a distinct enrichment of the TGF‐beta signaling pathway in high‐AD populations (Figure 2C). Differential expression (DE) analysis identified several known and potential new candidate genes that distinguished each population. Consistent with expectations, the high‐AD group exhibited increased expression of PPARG, WNT16, BMP5, CREB3L3, CEBPA, FABP4, and CXCR4 compared to the low‐AD group (Figure 2D). These genes are key early regulators of adipogenesis and the formation of mature adipocytes.^32^, ^33^ Interestingly, the RNA sequencing results of the relapse group closely resembled the gene expression levels of the high‐AD group of BM‐MSCs. Notably, we observed significant down‐regulation of Gremlin1 (GREM1) in the high‐AD group, suggesting its potential influence on adipogenic differentiation (Figure 2E).

### 4.4 Grem1 Deficiency promotes adipogenic differentiation of BM‐MSCs through the BMP‐SMAD pathway

**FIGURE 3:**
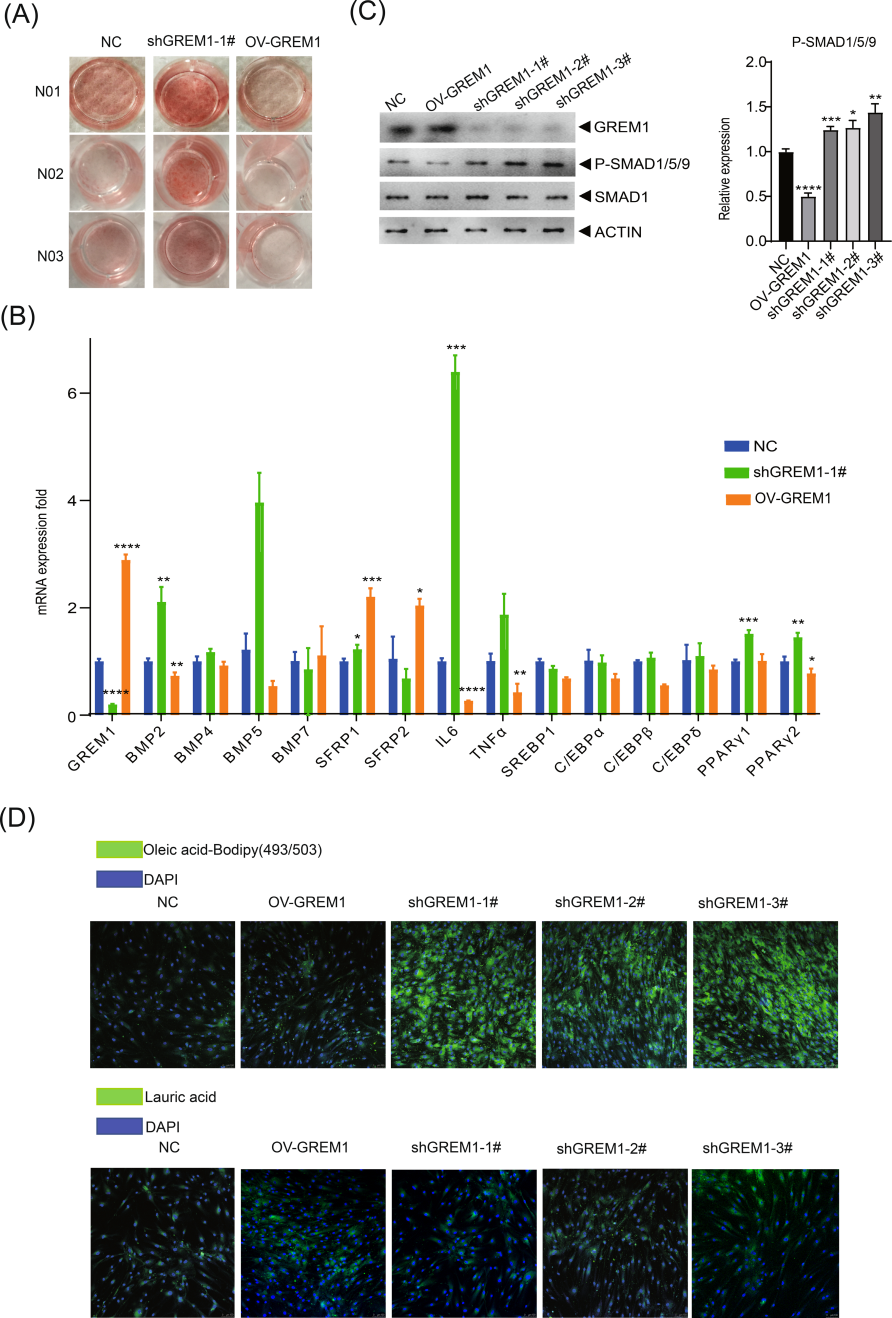
Deficiency of Gremlin‐1 promoted the adipogenic differentiation of BM‐MSCs. (A) The full field of view image of Oil Red O staining in NC‐MSCs, shGREM1‐MSCs‐1#, and ovGREM1‐MSCs at the end of adipogenic differentiation. (B) The gene expression levels of *GREM1* and other related genes that are involved in regulating adipogenic differentiation by real‐time quantitative PCR (RT‐PCR). ns *p* > 0.05, *0.01 < *p* < 0.05, **0.001 < *p* < 0.01, ***0.0001 < *p* < 0.001, and ****p < 0.0001. (C) WB experiment verifies the expression of GREM1 and the activation of the Smad signaling pathway. Grayscale values of proteins were evaluated by ImageJ. (D) Top: Fluorescence microscopy of BM‐MSCs in the presence of Oleic acid and stained with BODIPY 493/503, and bottom: Acid uptake assay was done with Screen Quest™ Fluorimetric Fatty Acid Uptake Assay Kit (AAT Bioquest) following the manufacturer's instructions.

GREM1 plays a crucial role in normal skeletal development and homeostasis.^34^ In this study, we manipulated the expression of *GREM1* in BM‐MSCs from three normal donors (N01, N02, and N03) and found that silencing *GREM1 led to an* increase in the adipogenic differentiation capacity (Figure 3A). To further investigate the underlying mechanisms, we examined the expression levels of *GREM1* and other related genes involved in regulating adipogenic differentiation using RT‐PCR. BMPs, which are classified into four subgroups based on sequence and functional similarities, have diverse biological functions. As a BMP antagonist, knocking down *GREM1* resulted in enhanced expression of BMP2, BMP4, and BMP5, with BMP2 showing the most significant increase (Figure 3B). This could be attributed to the preference of Grem1‐BMP‐2 complexes over other BMPs.^18^ IL‐6, a multifaceted cytokine, is known to play a crucial role in metabolic regulation. Recent studies have shown that IL6 also significantly contributes to lipid metabolic homeostasis.^35^ We observed a 6‐fold increase in IL6 expression in shGREM1 cells and its inhibition in overexpressing cells, which may have implications in adipogenesis. Furthermore, a slight but statistically significant upregulation of PPARG, a gene involved in adipogenic differentiation, was observed in shGREM1‐MSCs. Interestingly, the elevated expression of SFRP,^36^ a soluble WNT inhibitor, was also noted to coincide with Gremlin1 overexpression. To investigate the transmission of information by GREM1 to the adipogenic transcription cascade, we detected the expression level of proteins in the BMP/TGF‐β signaling pathway. In the shGREM1‐MSCs, we observed a significant increase in the expression level of P‐SMAD1/5/9 protein, but not SMAD1, compared to the NC‐MSCs (Figure 3C). Oleic acid is primarily stored as triacylglycerols, which serve as a neutral form of lipid storage and are the main accumulated free fatty acid (FFA) in ALL BM.^7^ The fluorescent neutral lipid dye (BODIPY493/503) is intrinsically lipophilic, similar to natural lipids, which enables it to facilitate quantification of neutral lipid content. Oleic acid can be stained positively by BODIPY for increased neutral lipid content. We detected the fatty acid absorption of oleic acid and lauric acid in *GREM1* knockdown and overexpression BM‐MSCs. We found a substantial increase in the accumulation of oleic acid in shGREM1 MSCs, while there was no difference in the ability to uptake lauric acid (LA) between groups (Figure 3D). This suggests that lauric acid, also known as systematically designated lauric acid, is typically used as a direct energy supply, while oleic acid is commonly stored in the form of lipid droplets.

### 4.5 The increasing oleic acid uptake promoted B‐ALL progression

**FIGURE 4:**
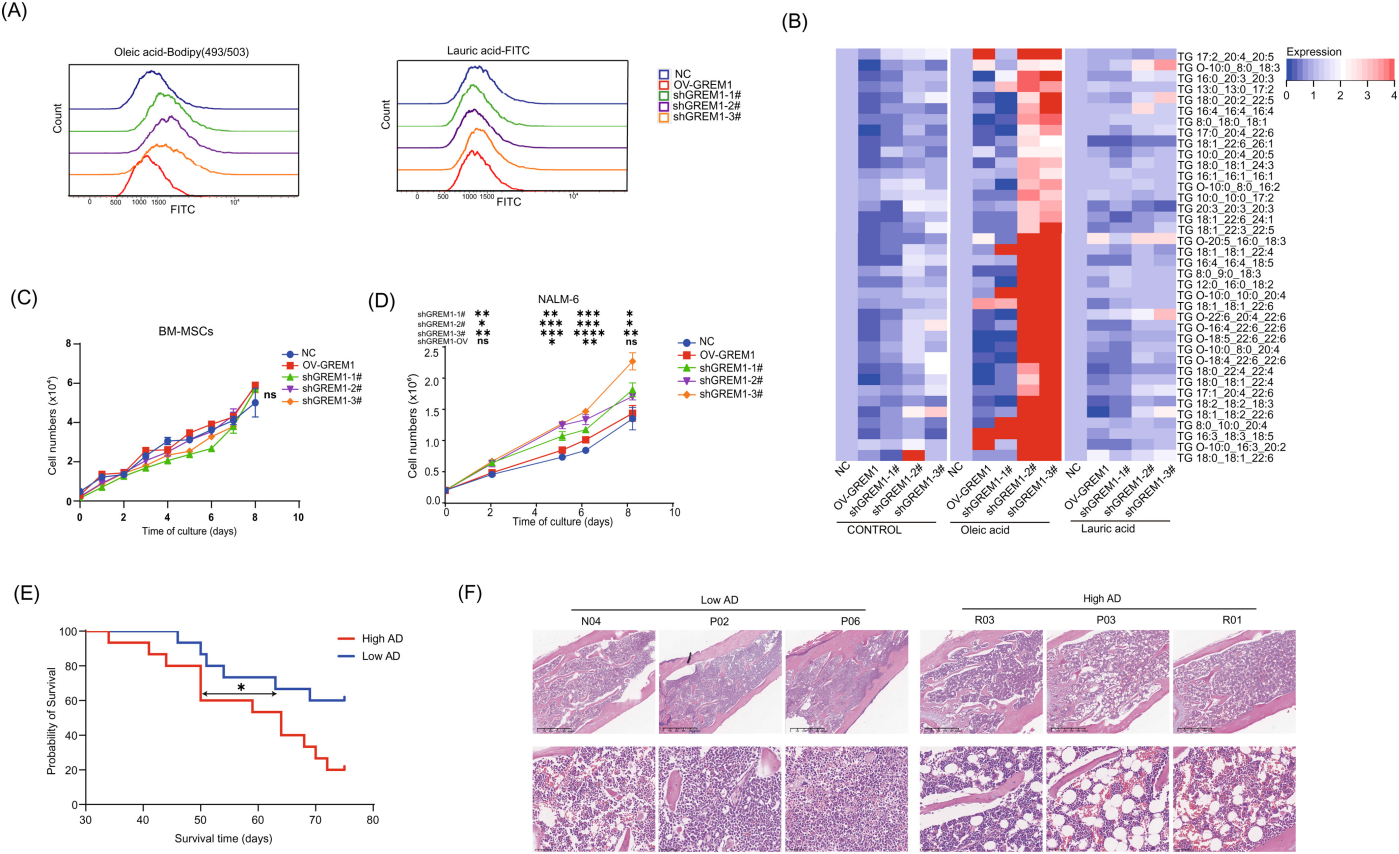
The increasing oleic acid uptake promoted B‐ALL progression. (A) Flow cytometry measurements for oleic acid(left) and lauric acid(right)uptake in NALM‐6 co‐cultured with NC‐MSCs, shGREM1‐MSCs, and ovGREM1‐MSCs. (B) LC–MS‐based metabolomics and lipidomics analyses of TG accumulation in NALM‐6 when co‐cultivated with culture supernatant of shGREM1‐MSC and treated with Oleic acid or lauric acid. (C, D) Growth curve analysis of NC‐MSCs, shGREM1‐MSCs, and ovGREM1‐MSCs (C), and NALM‐6 co‐cultured with NC‐MSCs, shGREM1‐MSCs, and ovGREM1‐MSCs (D). Comparisons were calculated using multiple unpaired *t* tests, ns *p* > 0.05, *0.01 < *p* < 0.05, **0.001 < *p* < 0.01, ***0.0001 < *p* < 0.001, and ****p < 0.0001. (E) Kaplan–Meier analyses of survival of mice engrafted with NALM‐6 and BM‐MSCs. High AD group showed a significantly effect on mouse survival, *0.01 < *p* < 0.05. (F) The top panels are images of the distal femurs stained with H&E (magnification ×4). The bottom panels are sections stained with H&E showing the numerous large adipocytes in bone marrow from distal femurs (magnification ×20).

We hypothesized that the accumulation of oleic acid in BM‐MSCs could potentially enhance the proliferation of leukemia cells. In order to investigate this hypothesis, we conducted a co‐culture experiment involving BM‐MSCs and the B‐ALL cell line NALM‐6. We observed an increase in the uptake of oleic acid by NALM‐6 when co‐cultured with shGREM1 MSC (Figure 4A). We then performed the LC–MS‐based metabolomics and lipidomics analyses to detect TG accumulation in NALM‐6 when co‐cultivated with the culture supernatant of shGREM1‐MSC and treated with Oleic acid, but not lauric acid (Figure 4B). This phenomenon was also confirmed in patient samples, where PBMCs isolated from patients with high‐AD BM‐MSCs showed a higher propensity for TG accumulation (Figure S3A). It is worth noting that P14, despite having low‐AD BM‐MSCs but with high accumulation of TG, was the only B‐ALL patient who died at diagnosis during treatment. These data indicate that abnormal triglyceride accumulation could be associated with the risk of death. Next, we observed no noticeable difference in cell proliferation between *GREM1* knockdown and overexpressed BM‐MSCs when cultured alone (Figure 4C). However, when co‐cultured with the B‐ALL cell line NALM‐6, shGREM1‐MSC significantly promoted leukemia cell proliferation in vitro (Figure 4D), indicating that fatty acid uptake plays a key role in leukemia proliferation. On the other hand, *GREM1* overexpression did not decrease the capacity for oleic acid absorption or the proliferation of NALM‐6.

In most cases, primary precursor‐B ALL cells undergo rapid apoptosis in vitro. Although the addition of cytokines and stromal feeder cells to cultures can prolong the survival of ALL cells for a few hours to days, only a few cases show ex vivo proliferation and expansion.^37^ Therefore, we examined the change in blast percentage of PBMC isolated from nine relapsed patients when co‐cultured with their own BM‐MSCs. The tumor blasts either increased or slightly decreased when co‐cultured with high AD BM‐MSCs, whereas a significant decrease in the percentage of tumor blasts was observed when co‐cultured with low AD BM‐MSCs (Table S3). These findings, obtained from both cell line and primary cell experiments, suggest that fatty acid uptake plays a crucial role in leukemia proliferation.

Furthermore, we investigated the impact of BM‐MSCs with varying levels of *GREM1* on tumor growth in vivo. Considering the negative correlation between GREM1 expression levels and adipogenic efficiency as depicted in Figures 1F,G and 2D, we classified BM‐MSCs with high GREM1 expression and low adipogenic efficiency into the ‘Low AD’ group, and those with low GREM1 expression and high adipogenic efficiency into the ‘High AD’ group. NALM‐6 cells were co‐implanted into immunocompromised mice through intravenous tail injection. As shown in Figure 4E, the high‐AD BM‐MSCs group (with low expression of GREM1) significantly reduced the survival rate of the animals (*p* = 0.0365), providing in vivo evidence supporting the role of GREM1 deficiency derived from BM‐MSCs in the progression of ALL. Additionally, we observed significant proliferation of fat cells in the bone marrow of the high‐AD groups (Figure 4F), which was believed to contribute to the disease progression and subsequent mortality.

### 4.6 Expression of GREM1 in BM‐MSCs can affect the drug sensitivity of leukemia cells

**FIGURE 5:**
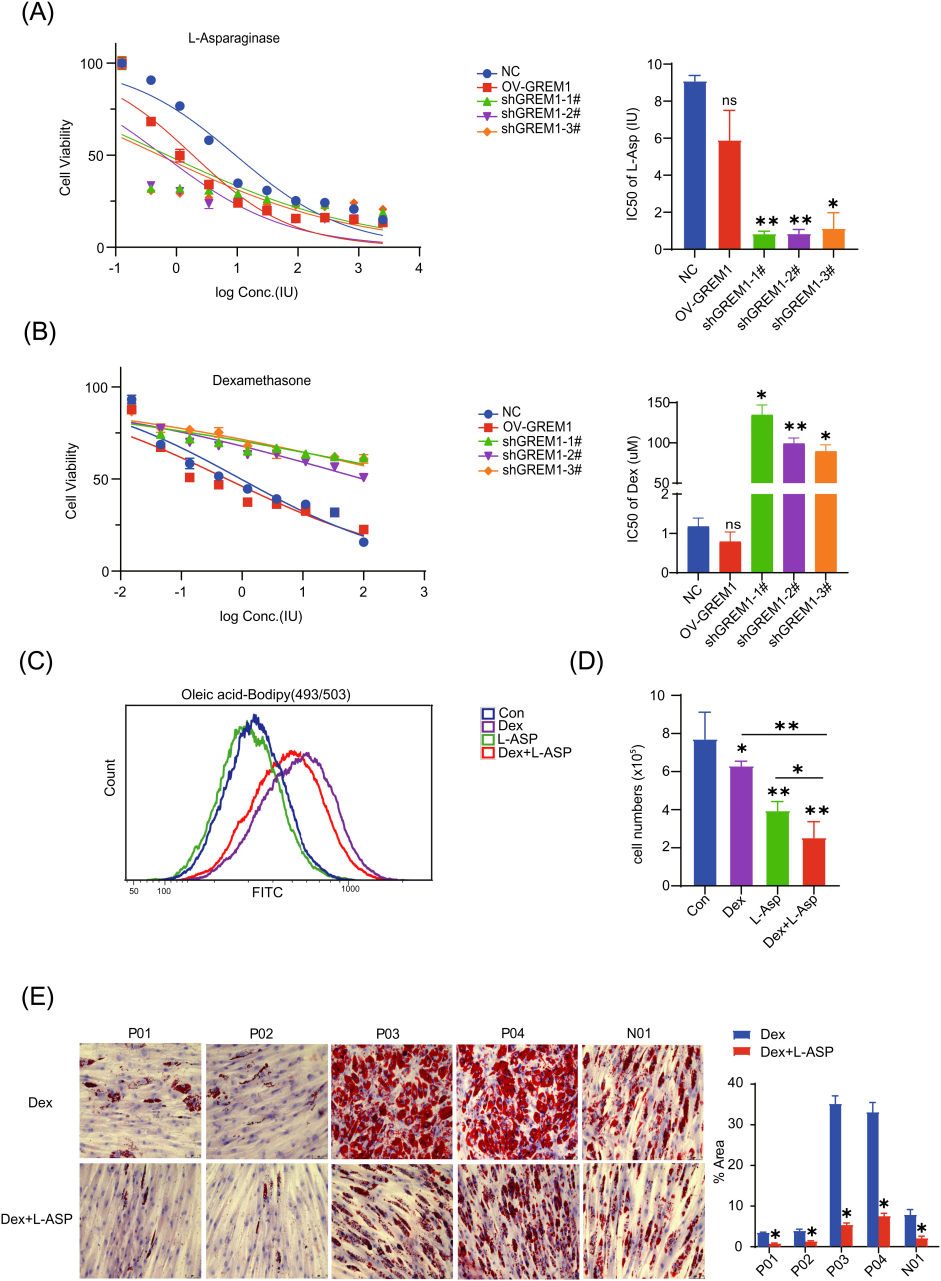
Expression of GREM1 in BM‐MSCs can affect the drug sensitivity of leukemia cells. (A,B) CCK‐8 assay showing that NALM‐6 co‐cultured with culture supernatant has an effect on L‐asparaginase (A) or dexamethasone (B) sensitivity and resistance induced by GREM1 expression of BM‐MSCs. Data represent mean ± SEM. ns *p* > 0.05, *0.01 < *p* < 0.05, **0.001 < *p* < 0.01. (C) Flow cytometry measurements for oleic acid uptake in NALM‐6 exposed to dexamethasone (1 μM) and L‐asparaginase (1 IU/mL), either in combination or alone. (D) The cell numbers of NALM‐6 exposed to dexamethasone (1 μM) and L‐asparaginase (1 IU/mL), either in combination or alone for 24 h. *0.01 < *p* < 0.05, **0.001 < *p* < 0.01. (E) L‐asparaginase significantly reduced the adipogenic differentiation of BM‐MSCs. Data represent mean ± SEM. *0.01 < *p* < 0.05.

Dexamethasone (Dex) and L‐asparaginase(L‐ASP) are commonly used chemotherapy drugs for B‐ALL and have a potent impact on lipid metabolism.^38^, ^39^ Co‐cultivating NALM‐6 cells with the culture supernatant of shGREM1‐MSC for 72 h markedly increased their sensitivity to L‐asparaginase, while they showed resistance to dexamethasone (Figure 5A,B). We further observed the drug effects on the ability of cells to uptake oleic acid. As shown in Figure 5C, there is an increase in the uptake of oleic acid by NALM‐6 when preincubated with dexamethasone alone or in combination with, but not L‐asparaginase alone. However, treatment with L‐asparaginase alone can significantly inhibit cell growth of NALM‐6 when co‐cultivated with the culture supernatant of shGREM1‐MSC for 24 h. Moreover, the combination of dexamethasone further inhibited cell growth compared to either treatment alone (Figure 5D). This result indicates that dexamethasone mainly affects an early step in lipid synthesis, potentially fatty acid uptake, impacting overall lipid production. Leukemia cells, particularly sensitive to asparagine deficiency, exhibit accelerated cell death when combined with the disruption of one or multiple pathways in maintaining lipid metabolism balance, including lipid uptake, synthesis, transport, and fatty acid oxidation. Dexamethasone is essential for the adipogenic induction of BM‐MSCs; without it, successful differentiation cannot be achieved. It is utilized at a concentration of 1 μM in the adipogenic differentiation medium. Conversely, the addition of L‐ASP resulted in a decrease in the differentiation potential of BM‐MSCs towards the adipogenic lineage (Figure 5E). These findings collectively demonstrate that shGREM1‐MSC support the growth of leukemia cells, but this effect can be inhibited by L‐asparaginase.

In summary, the bone marrow adipose niche and abnormal triglyceride accumulation in serum are both significant sources of extracellular lipids of leukemia. Deficiency of GREM1 induces adipogenicity of BM‐MSCs, leading to the rapid proliferation of leukemia cells. However, the efficiency of adipogenic differentiation can be inhibited by L‐Asparaginase to prevent drug resistance, relapse, and death.

## 5 DISCUSSION

The heterogeneity in adipogenic differentiation of B‐MSCs in B‐ALL was observed. Furthermore, a higher inclination towards adipogenic differentiation was observed in BM‐MSCs from relapsed patients. Subsequent research revealed that the high‐efficiency adipogenic differentiation of BM‐MSCs was consistent with the high level of TG in the serum. It was found that abnormal TG accumulation in serum (>2.26 mmol/L) is associated with drug resistance and a significantly increased risk of death in childhood B‐ALL. The deficiency of GREM1 induced adipogenicity through the BMP/SMAD pathway, thereby contributing to the proliferation of leukemia cells by increasing fatty acid uptake. However, this effect could be inhibited by L‐asparaginase. Our study on the interplay between leukemia cells and the tumor microenvironment provides new insights into understanding drug resistance and relapse, and offers a new avenue for therapeutic development in refractory/relapsed B‐ALL.

The bone marrow niche is an important source of extracellular lipids. The complexities of pathway interactions between adipogenic and osteogenic differentiation of BM‐MSCs can be confusing in different conditions. BM‐Ads are mainly derived from Bone marrow‐derived mesenchymal stem cells (BM‐MSCs). Factors involved in lipid‐induced differentiation of BM‐MSCs may affect the progression of leukemia. Notable signaling pathways such as BMP, Wnt, and Hippo have been implicated in the regulation of MSC differentiation.^40^, ^41^ PPARγ regulates both the terminal differentiation and metabolism in mature adipocytes^42^ and its activation renders leukemic stem cells, previously resistant to treatment, susceptible to targeted therapy in CML.^43^ The multiple myeloma‐derived secreted Wnt antagonists, SFRP, attenuate Wnt signaling in osteoblast precursors, impairing osteoblast differentiation and contributing to the development of osteolytic lesions.^44^ IL6 and TNFα are elevated in AML and/or MDS patients and are potential drivers of HSC dysfunction.^45^ Here, we identified GREM1 as a new regulator of MSC differentiation. Gremlin‐1 is a BMP antagonist and is well‐known for playing a cardinal role in organ formation and being associated with specific cancers and organ fibrosis^46^, ^47^ but it has rarely been studied in leukemia. We found that *GREM1* silencing increased the capacity of adipogenic differentiation of BM‐MSCs through the BMP/SMAD signaling pathway. The B‐ALL cell line NALM‐6 showed a higher proliferation rate when co‐cultured with shGREM1‐MSC. The BM‐MSCs isolated from P04 (with relatively low GREM1 expression) significantly decreased the survival of mice transplanted with B‐ALL cells. We assume that the relapse may arise from undetectable leukemia cells that survive in the bone marrow adipose microenvironment. Higher levels of TG in serum are associated with increased efficacy of adipogenicity in BM‐MSCs, leading to higher accumulation of TG in leukemia cells and ultimately resulting in higher mortality. Oleic acid is typically stored in the form of lipid droplets, whereas lauric acid (LA) is commonly utilized as a direct energy source. Due to their enhanced proliferation, leukemia cells require the uptake of extracellular lipids directly from the microenvironment to synthesize complex membrane lipids and provide energy. The analysis of exogenous lipid differences, which arise from the GREM1‐deficient BM‐MSCs, is essential for elucidating the role of differential lipid metabolites on B‐ALL cells. Furthermore, leukemia cells are believed to play a pivotal role in this lipid metabolism disorder. By conducting a transcriptome difference analysis of B‐ALL cells between high and low‐efficiency adipogenic differentiation groups, we can identify the differential genes that regulate the expression of gremlin‐1 in BM‐MSCs. Investigating the functions of these differential genes may provide a comprehensive understanding of the potential mechanisms.

Approximately 10% to 20% of patients experience relapse with chemotherapy‐resistant B‐ALL. Dexamethasone is commonly used as the initial chemotherapy treatment for leukemia, but it has several side effects including insulin resistance and effects on fat storage, liver fat, and lipid fuel fluxes.^38^, ^48^ L‐Asparaginase also disturbs the metabolic homeostasis of leukemic cells and can lead to allergic reactions, thrombosis, and acute pancreatitis.^49^, ^50^ In our co‐cultivation and drug sensitivity experiments, we were surprised to find that NALM‐6 cells were more sensitive to L‐asparaginase but more resistant to dexamethasone when co‐cultured with the supernatant of shGREM1‐MSC for 72 h. By measuring GREM1 expression in BM‐MSCs, we may be able to optimize drug selection and consider using L‐Asparaginase instead of dexamethasone for patients with low GREM1 expression in BM‐MSCs. This could potentially improve drug safety and efficacy, reducing the risk of drug resistance, relapse, and death. Given that acute pancreatitis‐associated adipose tissue has been found to undergo lipolysis even in the absence of adipocyte triglyceride lipase, and considering the recently reported important role of GREM1 in pancreatic cancer,^17^ we are particularly interested in exploring the relationship between hypertriglyceridemia‐induced pancreatitis and asparaginase‐induced pancreatitis. In future study, we intend to investigate the possibility of targeting Gremlin‐1 to prevent L‐asparaginase resistance and asparaginase‐induced pancreatitis.

The abnormalities of lipid metabolism in tumor cells which accelerate disease progression have been acknowledged by numerous researchers. Our research primarily focuses on the changes in extracellular lipids that drive leukemia development and disease relapse. We demonstrate a novel mechanism wherein GREM1 deficiency induces adipogenicity of BM‐MSCs, leading to the rapid proliferation of leukemia cells and a high risk of death after relapse. Therefore, GREM1 deficiency in BM‐MSCs can be considered a new adverse clinical prognostic factor. Intervening in the adipogenic differentiation of BM‐MSCs shows promise as a therapeutic strategy to prevent recurrence and improve survival rates in childhood B‐ALL relapse.

## AUTHOR CONTRIBUTIONS

**Lili Song:** Conceptualization; funding acquisition; project administration; validation; visualization; writing – original draft; writing – review and editing. **Rui Zhang:** Formal analysis; methodology. **Liya Pan:** Data curation. **Qiang Mi:** Data curation; resources. **Yi Yang:** Methodology. **Xiang Wang:** Resources. **Yani Ma:** Resources. **Shuhong Shen:** Resources. **Benshang Li:** Resources; supervision. **Yanxin Li:** Supervision; writing – review and editing. **Li Hong:** Data curation; funding acquisition; supervision; writing – review and editing.

## CONFLICT OF INTEREST STATEMENT

The authors declare no conflict of interest.

## ETHICS STATEMENT

The use of human samples in this study was approved by the Research Ethics Board at Shanghai Children's Medical Center (SCMC) with approval number SCMCIRB‐Y2019006. All patients gave written informed consent before inclusion in this study. All animal experiments were approved by Shanghai Children's Medical Center (SCMC) Experimental Animal Welfare Ethics Committee and were performed in accordance with the guidelines approved by Shanghai Jiao Tong University Institutional Animal Care and Use Committee (IACUC) (approval number SCMCIACUC‐K2019003).

## Supporting information


**DATA S1.** Supporting information.

**TABLE S5.** The summary of the sequencing coverage and quality statistics.

## Data Availability

All sequencing data are managed by the Genome Sequence Archive for Human (https://ngdc.cncb.ac.cn/gsa-human/browse/HRA003645) and are available upon reasonable request. The raw metabolomic data generated in this study have been uploaded to MetaboLights (http://www.ebi.ac.uk/metabolights/login) with identifier no. MTBLS10103. The lipid serum data from patients can be obtained from the corresponding author upon reasonable request. Additional relevant data supporting the findings of this study are available within the article and its Supplemental Information files. Further information is available from the corresponding author upon request.
