# Supplementary material for: GREM1 deficiency induced bone marrow adipose niche promotes B‐cell acute lymphoblastic leukemia disease progression
Source: Int J Cancer. 2025 Apr 26;157(3):559–72. doi: 10.1002/ijc.35418 (PMC12141984; doi:10.1002/ijc.35418)
Supplement: Supplementary file 1 — DATA S1. Supporting information. [file IJC-157-559-s002.pdf]

GREM1 Deficiency induced Bone Marrow Adipose Niche promotes B-cell Acute Lymphoblastic Leukemia Disease Progression

Lili Song, Rui Zhang, Liya Pan, Qiang Mi, Yi Yang, Xiang Wang, Yani Ma, Shuhong Shen, Benshang Li, Yanxin Li and Li Hong

Contents:

Supplemental Table 1-4: Page 2-4

Supplemental Table 5: It is available in separate excel file.

Supplemental Figure 1-3: Page 5-7

Supplemental Table 1. Correlation between clinical characteristics and the TG level in childhood B-ALL

Variable		TG≤2.26mmol/L n=150	TG>2.26mmol/L n=95	*P* value
Age at diagnosis (y)				
	0 to 9	131 (87.33%)	77 (81.05%)	0.1810
	≥9	19 (12.67%)	18 (18.95%)	
Gender				
	Female	57 (38.00%)	39 (41.05%)	0.6334
	Male	93 (62.00%)	56 (58.95%)	
WBC at diagnosis(x10^9/L)				
	<50	133(88.67%)	81(85.26%)	0.4349
	≥50	17(11.33%)	14(14.74%)	
Blasts (%)				
	<80	65 (43.33%)	41 (43.16%)	0.9785
	≥80	85 (56.66%)	54 (56.84%)	
Genetic variation				
	TEL-AML1	39(26.00%)	13(13.68%)	**0.0216**
	BCL-ABL	9(6.00%)	5(5.26%)	0.9678
	TCF3-PBX1	3(2.00%)	3(3.16%)	0.8830
	MLL rearrangement	2(1.33%)	4(4.21%)	0.3195
Risk group				
	Low	92(61.33%)	55(57.89%)	0.5924
	Intermediate	58(38.67%)	37(38.95)	0.9650
	High	0(0.00%)	3(3.16%)	0.0572
MRD				
	D19≥0.1%	57 (38.00%)	33 (34.74%)	0.6057
	D46≥0.01%	15 (10.00%)	15 (15.79%)	0.1780
Treatment protocol				
	CCCG-ALL-2015	106(70.67%)	66(69.47%)	0.8423
	CCCG-ALL-2020	44(29.33%)	29(30.53%)	
Outcome				0.7213
	CR	11 (7.33%)	9 (9.47%)	0.1659
	Relapse	11 (7.33%)	9 (9.47%)	0.7213
	Death	1 (0.67%)	5 (5.26%)	**0.0339**

Supplemental Table 2. Multivariate logistic regression analysis of risk factors for death

Variable	Multivariate			
	OR	95% CI (profile likelihood)	|Z|	*P* value
Gender	1.888	0.1556 to 21.90	0.5346	0.5929
Age at diagnosis (y)	1.183	0.8243 to 1.665	1.024	0.3058
TG level	1.485	0.9319 to 2.231	2.082	**0.0374**
MRD(D19)	7.888	2.897e-009 to 45031	0.3160	0.7520
Infection	27.33	2.341 to 539.1	2.521	**0.0117**
Treatment related toxicity	3.901	0.2358 to 49.52	1.064	0.2872
Extramedullary infiltration	8.260	0.1934 to 377.2	1.187	0.2354
Relapse	14.71	0.7061 to 375.8	1.848	0.0646

Supplemental Table 3. The change in blast percentage after PBMC co-cultured with BM-MSCs

Group	Patient ID	Blasts at relapse (%)	Blasts after co-culture (%)	Adipogenic efficiency (%)
Relapsed	R16	60	14.28	0.74
Relapsed	R07	21	0.01	4.97
Relapsed	R21	15	0.21	7.60
Relapsed	R09	79	87.01	12.11
Relapsed	R14	29	19.62	24.57
Relapsed	R12	6	66.67	26.55
Relapsed	R15	28	22.27	32.97
Relapsed	R13	9	6.75	39.89

Supplemental Table 4. Primers for RT-PCR

Gene name	Forward primer（5'→3’）	Reverse primer（5'→3’）
β-actin	CATCCGCAAAGACCTGTACG	CCTGCTTGCTGATCCACATC
GREM1	AGGCCCAGCACAATGACTCAG	GTCTCGCTTCAGGTATTTGCG
PPARγ1	CTTTATGGAGCCCAAGTTTGAGTT	GGCTTCACATTCAGCAAACCT
PPARγ2	GGGTGAAACTCTGGGAGATTCTC	GATGCCATTCTGGCCCAC
C/EBPα	TTGGTCAAGGCCATGGGCA	TGCAGCTCTGGAGGAAGC
C/EBPβ	GCGCGAGCGCAACAACATC	TGCTTGAACAAGTTCCGCAG

C/EBPδ	CCTCCCAAAATGCTGGGATTAC	TTCCAGGTCTACGGAAGCAGTG
SREBP1	CCTTGCATTTTCTGACACGCT	TCCCCATCCACGAAGAAACG
IL6	AGACAGCCACTCACCTCTTCAG	TTCTGCCAGTGCCTCTTTGCTG
BMP2	TGTATCGCAGGCACTCAGGTCA	CCACTCGTTTCTGGTAGTTCTTC
BMP4	CTGGTCTTGAGTATCCTGAGCG	TCACCTCGTTCTCAGGGATGCT
BMP5	CTCTCATCAGGACTCCTCCAGA	GGAAGCTCACATAGAGTTCGTGC
BMP7	GAGTGTGCCTTCCCTCTGAACT	AGGACGGAGATGGCATTGAGCT
SFRP1	CAATGCCACCGAAGCCTCCAAG	CAAACTCGCTGGCACAGAGATG
SFRP2	CTCCAAAGGTATGTGAAGCCTGC	CCAGGATGATTTTGGTATCTCGG
CXCR4	CTCCTCTTTGTCATCACGCTTCC	GGATGAGGACACTGCTGTAGAG
CXCL12	CTCAACACTCCAAACTGTGCCC	CTCCAGGTACTCCTGAATCCAC
ROBO2	AAGGAGTGGACCACAGGCAAGT	CTCGGTAGCCTTGGATAAACTGG
IL8	GAGAGTGATTGAGAGTGGACCAC	CACAACCCTCTGCACCCAGTTT
IL2	AGAACTCAAACCTCTGGAGGAAG	GCTGTCTCATCAGCATATTCACAC
CSF3	TCCAGGAGAAGCTGGTGAGTGA	CGCTATGGAGTTGGCTCAAGCA
CD36	CAGGTCAACCTATTGGTCAAGCC	GCCTTCTCATCACCAATGGTCC
TNFα	TTCTGCCAGTGCCTCTTTGCTG	ATGGGCTACAGGCTTGTCACTC

Supplemental Figure 1

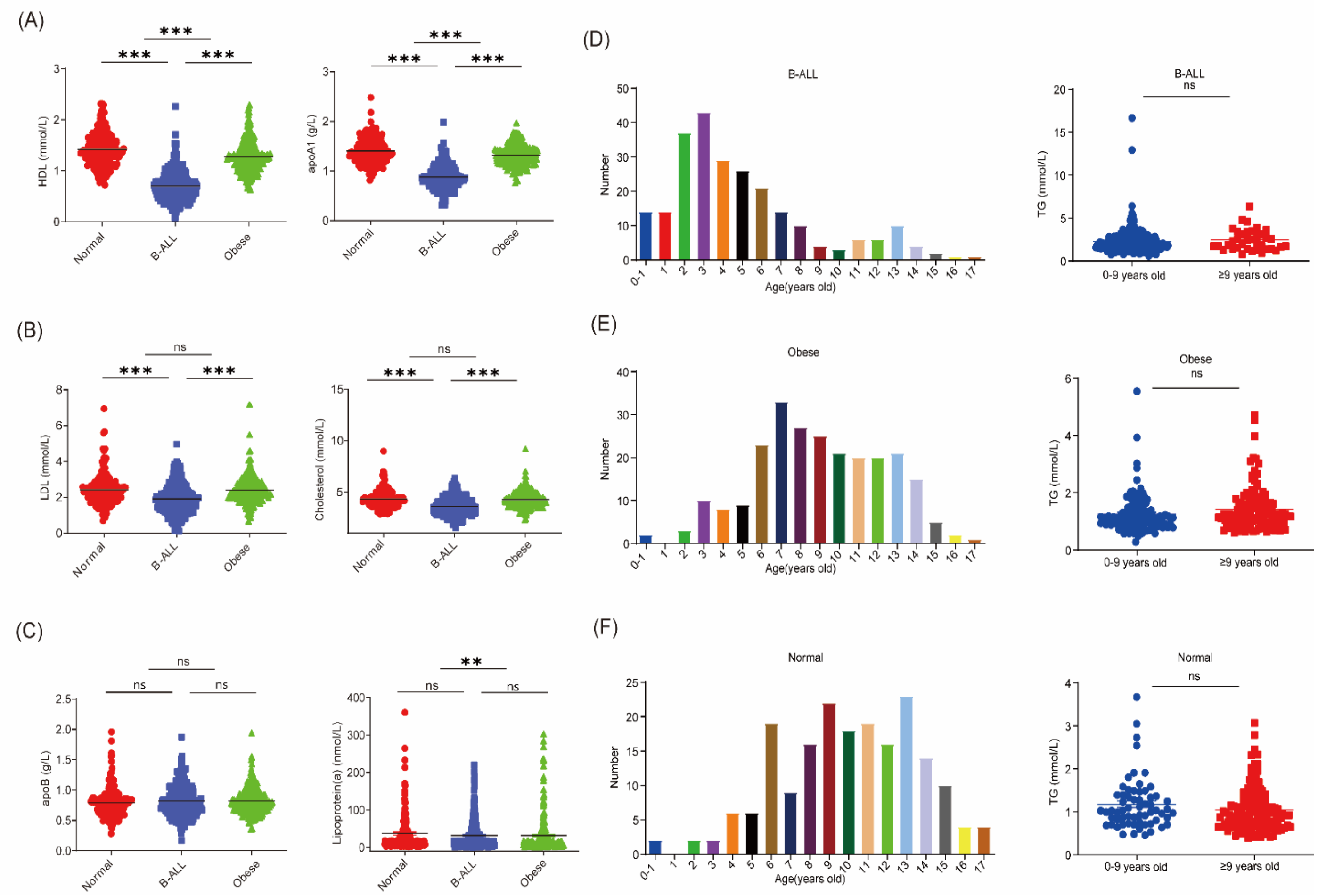

Supplemental Figure 1 Dyslipidemia in childhood B-ALL.

A-C, Scatter plot of HDL (mmol/L), apoA1(g/L), LDL (mmol/L), total cholesterol (mmol/L), apoB (g/L) and lipoprotein(a) (mmol/L) in normal group, B-ALL and obese patients. The figures were made using GraphPad Prism 9.0. Comparisons were calculated using two-sided Mann–Whitney tests, ns P>0.05, * 0.01<P<0.05, ** 0.001<P<0.01, *** 0.0001< P<0.001 and ****P<0.0001. D-F, Left: age distribution in normal group, B-ALL and obese patients. Right: Scatter plot of serum triglycerides (mmol/L) according to the age distribution of two subjects (0-9 years old and ≥9 years old) in normal group, B-ALL and obese patients, Comparisons were calculated using two-sided Mann–Whitney tests, ns P>0.05.

Supplemental Figure 2

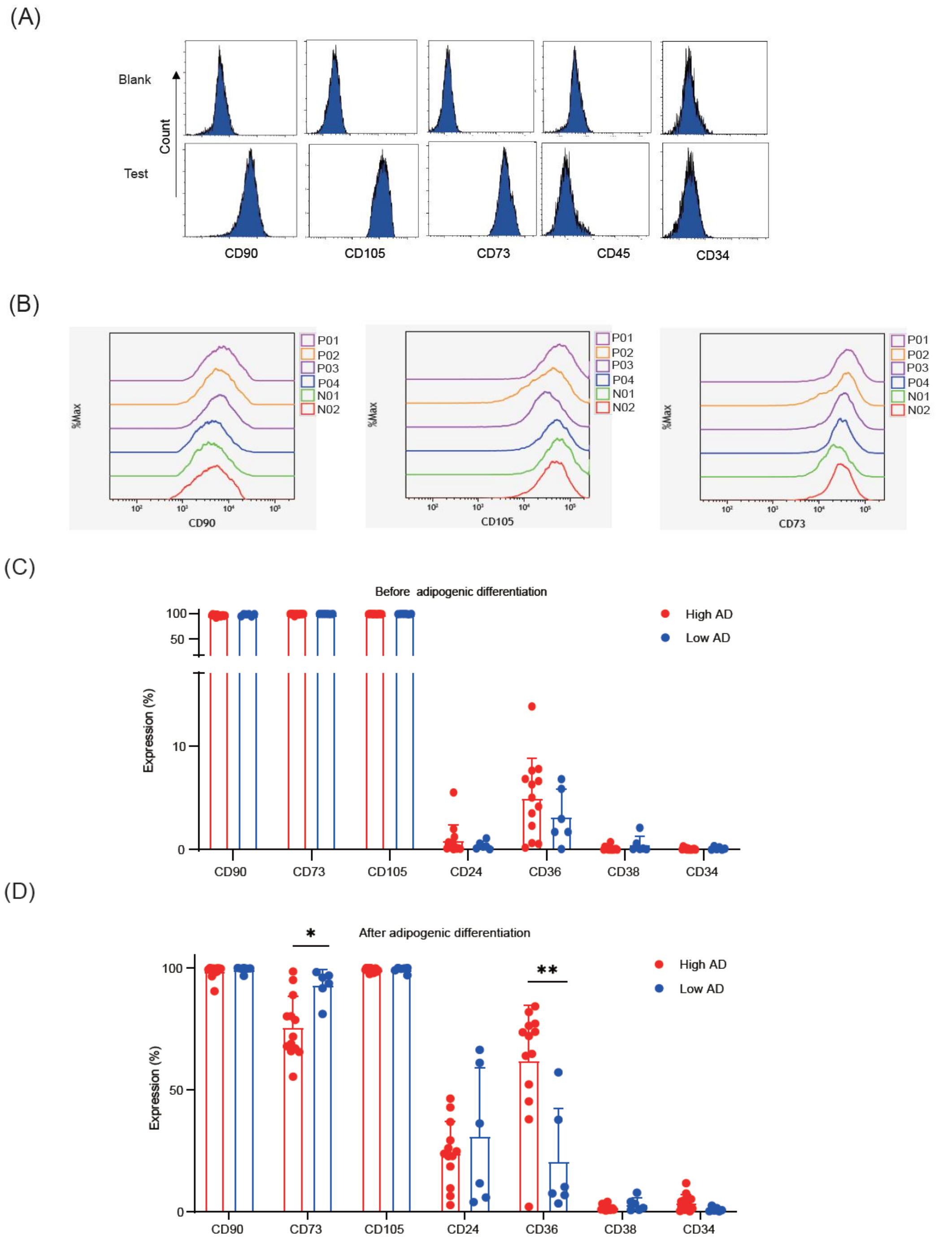

Supplemental Figure 2 MSC defining markers between ND-MSCs and B-ALL-MSCs

A-B, Expression of surface antigen CD73/CD90/CD105 in BM-MSCs were detected by flow cytometry, and peak shifts in fluorescence were analyzed using Kaluza software and it indicated no significant change in different patients. C, Expression of surface antigen CD73/CD90/CD105/CD24/CD36/CD38/CD34 in BM-MSCs at baseline were detected by flow cytometry and analyzed using Kaluza software. D, Expression of surface antigen CD73/CD90/CD105/CD24/CD36/CD38/CD34 in BM-MSCs at baseline were detected by flow cytometry and analyzed using Kaluza software. Comparisons were calculated using multiple unpaired t tests, * 0.01<P<0.05, ** 0.001<P<0.01.

Supplemental Figure 3

(A)

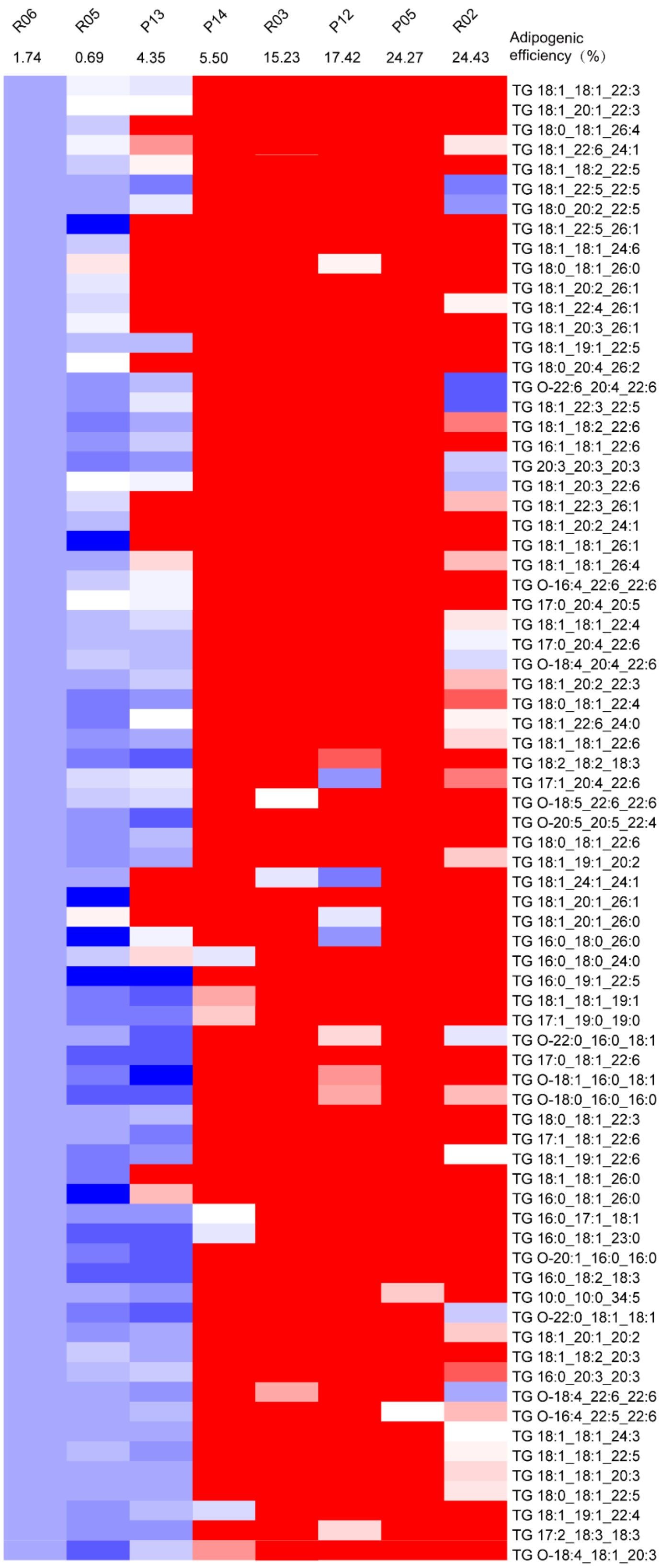

Supplemental Figure 3 Lipidomics analyses

A, LC-MS-based metabolomics and lipidomics analyses of TG accumulation in PBMC isolated from patients.
